# Analysis of Circulating MicroRNAs *In Vivo* following Administration of Dexamethasone and Adrenocorticotropin

**DOI:** 10.1155/2015/589230

**Published:** 2015-06-16

**Authors:** Ivan Igaz, Gábor Nyírő, Zoltán Nagy, Henriett Butz, Zsolt Nagy, Pál Perge, Peter Sahin, Miklós Tóth, Károly Rácz, Peter Igaz, Attila Patócs

**Affiliations:** ^1^Department of Gastroenterology, Szent Imre Teaching Hospital, Tétényi Street 12-16, Budapest 1115, Hungary; ^2^Molecular Medicine Research Group, Hungarian Academy of Sciences and Semmelweis University, Szentkirályi Street 46, Budapest 1088, Hungary; ^3^2nd Department of Medicine, Faculty of Medicine, Semmelweis University, Szentkirályi Street 46, Budapest 1088, Hungary; ^4^“Lendület-2013” Hereditary Endocrine Tumors Research Group, Hungarian Academy of Sciences and Semmelweis University, Szentkirályi Street 46, Budapest 1088, Hungary

## Abstract

*Purpose*. The interaction of hormones of the pituitary-adrenal axis and adrenal cortex-associated circulating microRNAs is mostly unknown. We have studied the effects of dexamethasone and adrenocorticotropin on the expression of five circulating microRNAs (*hsa-miR-27a, hsa-miR-200b, hsa-miR-214, hsa-miR-483-5p, and hsa-miR-503*) reported to be related to the adrenal cortex in plasma samples. *Methods*. Expression of microRNAs was studied in plasma samples of 10 individuals examined by 1 mg dexamethasone suppression test and another 10 individuals stimulated by 250 *μ*g tetracosactide (adrenocorticotropin). Total RNA was isolated and microRNA expression was analyzed by real-time reverse transcription quantitative polymerase chain reaction normalized to *cel-miR-39* as reference. *Results*. Only circulating *hsa-miR-27a* proved to be significantly modulated *in vivo* by hormonal treatments: its expression was upregulated by dexamethasone whereas it was suppressed by adrenocorticotropin. Secreted *hsa-miR-27a* was significantly induced by dexamethasone *in vitro* in NCI-H295R cells, as well. The expression of *hsa-miR-483-5p* proposed as diagnostic marker for adrenocortical malignancy was not affected by dexamethasone or tetracosactide administration. *Conclusions*. *hsa-miR-27a* expression is modulated by hormones of the hypothalamic-pituitary-adrenal axis both *in vitro* and *in vivo*. The biological relevance of *hsa-miR-27a* modulation by hormones is unclear, but the responsiveness of circulating microRNAs to hormones of the pituitary-adrenal axis is noteworthy.

## 1. Introduction

MicroRNAs (miRNA, miR) are short nonprotein coding RNA molecules involved in the posttranscriptional regulation of gene expression as parts of the epigenetic machinery. MicroRNAs were shown to be implicated in the regulation of several basic homeostatic processes like cell proliferation, apoptosis, development, immune regulation, hormone secretion, and so forth, [1]. Alterations of tissue microRNA profiles have been described in a wide array of diseases, for example, atherosclerosis, inflammatory diseases, and tumors [1–3]. Beside tissue microRNAs, novel data show that microRNAs are released in the circulation by three main mechanisms: (i) passive release from damaged cells (inflammation, necrosis), or (ii) active release packed in membrane vesicles (microvesicles, exosomes, and apoptotic bodies), or (iii) active release in complex with macromolecules like high density lipoprotein or Argonaute proteins [4]. The physiological function of circulating microRNAs is mostly unknown, but it is hypothesized that they might act as hormones conveying epigenetic information to distant tissues [5].

There are some data that the expression of tissue microRNAs is affected by hormones. Tissue microRNA profiles of steroid-producing organs have been shown to be modulated by treatment with hormones, for example, adrenocorticotropin (ACTH), dexamethasone, and estradiol [6, 7]. There are also findings showing that circulating microRNA levels might also be influenced by hormone actions [8]. To the best of our knowledge, the circulating microRNA levels after administration of dexamethasone and ACTH affecting the hypothalamic-pituitary-adrenal axis have not been studied in humans* in vivo*, yet.

Adrenocortical cancer (ACC) is a rare tumor with an incidence of 0.5–2/million/year. The preoperative diagnosis of malignancy in adrenal tumors is very difficult. It is rather difficult to establish malignancy in small tumors and to exclude it in large adrenal tumors. Some circulating microRNA biomarkers, including* hsa-miR-483-5p,* have been proposed as promising markers of malignancy in ACC [9–11].

Keeping in mind that during the evaluation of an adrenal mass dexamethasone suppression and ACTH stimulation tests are widely used diagnostic approaches, the question might be raised whether the expression level of circulating microRNAs including proposed biomarkers for adrenocortical malignancies is affected during these functional endocrine tests.

Our objective has been to study whether the expression of selected circulating microRNAs is affected by dexamethasone and ACTH administration* in vivo* in plasma samples of humans. We have included microRNAs used in the diagnosis of adrenocortical cancer to assess whether their plasma levels are affected by these treatments.

We have selected five microRNAs (*hsa-miR-27a, hsa-miR-200b, hsa-miR-214, hsa-miR-483-5p,* and* hsa-miR-503*) whose tissue counterparts were shown to be modulated by ACTH or dexamethasone in an animal model (*hsa-miR-27a, hsa-miR-200b, hsa-miR-214,* and* hsa-miR-503*) [7] and/or involved in the pathogenesis of ACC (*hsa-miR-214, hsa-miR-483-5p,* and* hsa-miR-503*) [9–11].* hsa-miR-483-5p* is overexpressed not only in the tissue of adrenal cancer but also as a circulating microRNA in patient's blood [9–11]. We have studied these selected microRNAs in altogether 20 individuals examined for hypercortisolism (Cushing's syndrome) by low-dose dexamethasone test [12] and for adrenal insufficiency or late onset congenital adrenal hyperplasia (21-hydroxylase deficiency) by ACTH (tetracosactide) test [13].

## 2. Subjects and Methods

### 2.1. Patients

10 patients were tested for suspected hypercortisolism by low-dose overnight (1 mg) dexamethasone suppression test suffering from obesity, hirsutism, hypertension, and adrenal incidentaloma. Another 10 patients have been examined by 250 *μ*g tetracosactide (Cosyntropin, Sandoz Inc.) for suspected Addison's disease or late onset congenital adrenal hyperplasia (deficiency of 21-hydroxylase) suffering from weakness, secondary oligomenorrhea, infertility, or hirsutism. Baseline cortisol was taken between 7.00 and 9.00 a.m. in fasting condition. Dexamethasone was taken at 11.00 pm, and blood was drawn the next morning between 7.00 and 9.00 a.m. Blood was taken one hour after tetracosactide administration. Patient data are included in Table 1. All tested individuals have been eventually found to be free from any functional disturbance of the hypothalamic-pituitary-adrenal axis. The study was approved by the Ethical Committee of the Hungarian Health Council and informed written consent was obtained from all patients involved.

### 2.2. RNA Isolation and Real-Time Reverse Transcription Quantitative Polymerase Chain Reaction (RT-qPCR) from Plasma Samples

RNA isolation has been performed as described in our previous study [10]. Briefly, EDTA-anticoagulated blood was taken from patients and centrifuged at 3000 rpm for 20 minutes at 4°C. All extracted plasma samples were stored at −80°C until further processing.

Total RNA was isolated from 200 *μ*L plasma with Qiagen miRNeasy Mini Kit (Qiagen GmbH, Hilden, Germany) according to the manufacturer's protocol with minor modifications, as described earlier [10]. RNA concentration was measured by NanoDrop 1000 Spectrophotometer (Thermo Fisher Scientific Inc., Waltham, MA, USA), and the quality and quantity were determined by an Agilent 2100 Bioanalyzer (Agilent Tech. Inc., Santa Clara, CA, USA). RNA Integrity (RIN) numbers of RNA isolated from plasma samples were low (around 2.0), that is, similar to reported findings on RNA isolated from blood [14]. RNA was stored at −80°C until use.

10 ng of total RNA was reverse transcribed with TaqMan MicroRNA Reverse Transcription Kit (Applied Biosystems, Foster City, CA, USA) and the specific looped RT primer. RT-qPCR was performed by TaqMan Fast Universal PCR Master Mix (2x) (Applied Biosystems) on a 7500 Fast Real-Time PCR System (Applied Biosystems) according to the manufacturer's protocol. The following probes have been used:* hsa-miR-27a* (000408),* hsa-miR-200b* (002251),* hsa-miR-214* (002306),* hsa-miR-483-5p* (002338),* hsa-miR-503* (001048), and* cel-miR-39* (000200) as reference gene [15]. Samples were run in triplicate.

### 2.3. *In Vitro* Treatment of NCI-H295R Cells with Dexamethasone

The NCI-H295R adrenocortical carcinoma cell line was purchased from the American Type Culture Collection and maintained in the recommended media. For treatments, hormone-free fetal bovine serum (FBS) was prepared as follows: 0.1 g dextran coated charcoal (C6241, Sigma-Aldrich, St. Louis, MO) was added to 6 mL FBS and incubated for 24 h at 4°C. Then the mixture was centrifuged at 3000 ×g for 10 min and the supernatant was filtered through a 0.22 *μ*m filter. Cells were seeded on 6-well plates as 10^6^ cells/well using media containing 2.5% hormone-free FBS. Next day, cells were synchronized by serum starvation for 24 h. On the following day, 2.5% hormone-free FBS was added in the presence of 100 nM dexamethasone or vehicle (DMSO). After 8 h incubation, cells and supernatans were harvested and total RNA was extracted. Dexamethasone treatments were repeated four times.

Total RNA was extracted using miRNeasy Mini Kit (Qiagen) both from cells and culture medium according to the manufacturer's protocol with minor modifications, as described earlier [16]. RNA concentration was measured by NanoDrop 1000 Spectrophotometer (Thermo Fisher Scientific Inc.). RIN numbers determined by an Agilent 2100 Bioanalyzer (Agilent Tech. Inc., Santa Clara, CA, USA) varied between 9.0 and 10.0. RNA was stored at −80°C until use. RT-qPCR reactions were performed by Taqman miRNA Assays (Applied Biosystems) using specific primer/probe combinations:* hsa-miR-27a* (000408) and* cel-miR-39* (000200) as reference gene [15].

### 2.4. Statistical Analysis

To identify microRNAs showing significant expression changes, Student's *t*-test or Mann-Whitney *U* test was used depending on the results of Shapiro-Wilks normality test [10]. Data were expressed as ΔCt; thus higher ΔCt indicates lower expression, whereas lower ΔCt shows higher expression. Statistical analysis of RT-qPCR data was done by Statistica 7.0 (StatSoft Inc., Tulsa, OK, USA) software.

## 3. Results

### 3.1. Expression of Circulating MicroRNAs in Dexamethasone and ACTH Stimulation Tests* In Vivo*

**Figure 1:**
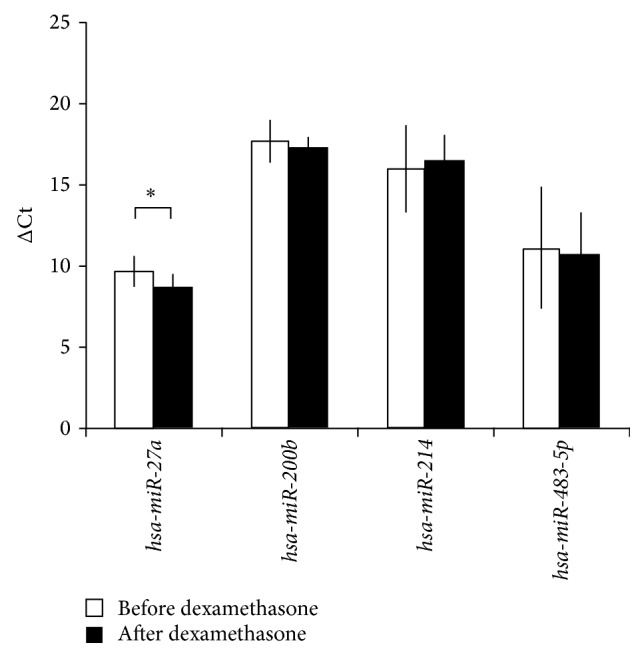
Expression change of microRNAs in plasma after 1 mg overnight dexamethasone test normalized to* cel-mir-39*. ΔCt values are represented: increased ΔCt indicates lower expression, whereas decreased ΔCt indicates higher expression (mean ± SD). ^*∗*^ *p* < 0.05, *n* = 10. *t*-test was performed following the Shapiro-Wilks normality test.

**Figure 2:**
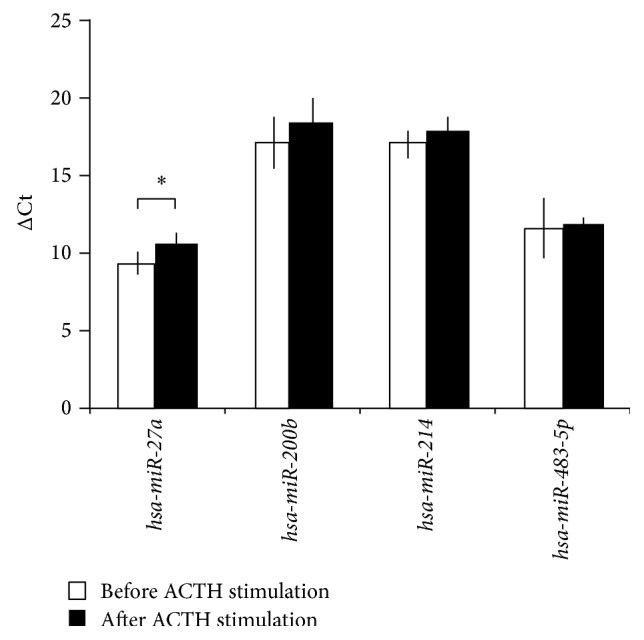
Expression change of microRNAs in plasma after 250 *μ*g tetracosactide test, normalized to* cel-mir-39*. ΔCt values are represented: increased ΔCt indicates lower expression, whereas decreased ΔCt indicates higher expression (mean ± SD). ^*∗*^ *p* < 0.05, *n* = 10. Results of Mann-Whitney *U* test.

From the five microRNAs selected, only one circulating microRNA,* hsa-miR-27a,* turned out to be significantly modulated by dexamethasone and tetracosactide treatment in our study. Most interestingly, dexamethasone and tetracosactide treatments resulted in opposite changes of* hsa-miR-27a* expression as dexamethasone upregulated its plasma level, whereas tetracosactide suppressed its expression (Figures 1 and 2).

The expression of* hsa-miR-503* proved to be so low in the plasma samples that we have decided to exclude it from further analysis (data not shown). We have not found any correlation between the changes of cortisol levels and circulating microRNAs neither in the dexamethasone nor in the tetracosactide tests (data not shown). There has been no correlation between basal* hsa-miR-27* levels and body weight either.

**Figure 3:**
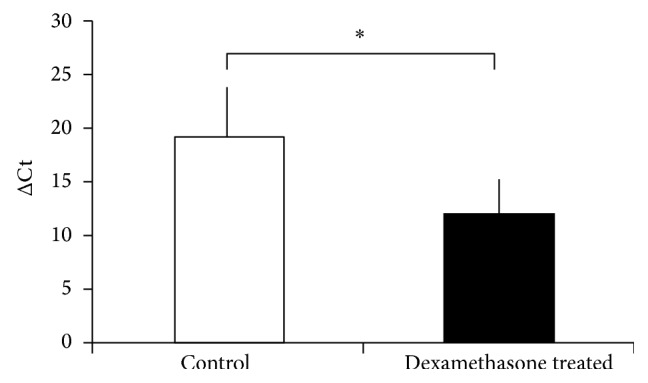
Expression change of secreted* hsa-miR-27a* after 100 nM dexamethasone treatment for 8 hours in the NCI-H295R adrenocortical cell line mediums normalized to* cel-mir-39*. ΔCt values are represented: increased ΔCt indicates lower expression, whereas decreased ΔCt indicates higher expression (mean ± SD). ^*∗*^ *p* < 0.05, *n* = 4. Results of *t*-test.

To confirm the dexamethasone responsiveness of* hsa-miR-27a*, we have performed* in vitro* experiments on the adrenocortical NCI-H295R cell line. We have observed dexamethasone responsiveness* in vitro*, as well. Dexamethasone significantly induced secreted* hsa-miR-27a* expression in NCI-H295R culture medium (Figure 3). Dexamethasone induced intracellular* hsa-miR-27a* in NCI-H295R cells too, but this has not reached statistical significance (data not shown). These results demonstrate that* hsa-miR-27a* is secreted by NCI-H295R cells and the level of secreted* hsa-miR-27a* is induced by dexamethasone, as well.

## 4. Discussion

We have found that the expression of circulating* hsa-miR-27a* is modulated by hormonal treatments* in vivo* in humans, as its expression is induced by dexamethasone and suppressed by ACTH. Dexamethasone induced secreted* hsa-miR-27a in vitro*, as well. The expression of the most promising circulating microRNA marker of adrenocortical malignancy,* hsa-miR-483-5p,* was not affected by these treatments also used as diagnostic tests supporting its applicability as a biomarker.

Circulating microRNAs are promising biomarkers in several diseases including tumors and atherosclerosis [17]. There are some data that their levels might be affected by hormonal changes, for example, in patients suffering from polycystic ovarian syndrome, the serum concentration of four microRNAs appeared to be in part correlated with serum free testosterone concentration [8].

To the best of our knowledge, the association of circulating microRNAs and the hormonal actions affecting the hypothalamic-pituitary-adrenal axis* in vivo* has not been explored in humans, yet. Some circulating microRNAs have been proposed as useful biomarkers for prediction of malignancy of adrenocortical tumors [9–11]. Since adrenocortical cancer is frequently associated with adrenocortical hormone overproduction [18, 19], the potential association of hypothalamic-pituitary-adrenal axis functioning and circulating microRNA levels might also be of interest. However, no data about the expression changes of circulating microRNAs during dexamethasone or ACTH-tests have been presented to date.

We have selected five circulating microRNAs for studying their responsiveness to dexamethasone and ACTH administration* in vivo*. Among these,* hsa-miR-214, hsa-miR-503,* and* hsa-miR-483-5p* have been proposed as tissue biomarkers for adrenocortical malignancy [20–22], and* hsa-miR-483-5p* has been found to be significantly overexpressed in blood samples of adrenocortical cancer patients, as well [9–11]. Moreover, the tissue expression of* miR-214* and* miR-503* was downregulated by ACTH in a rat model [7]. Two further microRNAs reported to be responsive to hormonal treatments in a rat model were included in our study: the expression of both* hsa-miR-27a* and* hsa-miR-200b* was shown to be downregulated by dexamethasone, whereas ACTH also downregulated* hsa-miR-27a* in rat adrenals [7].

The expression of the five selected circulating microRNAs has been studied* in vivo* using plasma samples of ten patients before and after low-dose dexamethasone testing and samples from ten patients before and after tetracosactide administration. All individuals included turned out to be eventually free from any functional abnormality of the hypothalamic-pituitary-adrenal axis.

From the microRNAs selected, only* hsa-miR-27* turned out to be significantly modulated by hormonal treatments* in vivo*. The expression of the other four circulating microRNAs that were shown to be associated with the adrenal was not affected by dexamethasone or ACTH-administration. The stable expression of circulating* hsa-miR-483-5p* not affected by these hormones supports its applicability as a biomarker of adrenocortical cancer. Our study has certainly limitations, since healthy individuals were tested, and a different response in ACC patients cannot be excluded.

Dexamethasone and tetracosactide treatments resulted in opposite changes of* hsa-miR-27a* expression as dexamethasone upregulated its plasma levels, whereas tetracosactide suppressed its expression (Figures 1 and 2). In addition, secreted* hsa-miR-27a* was significantly induced by dexamethasone treatment* in vitro* in the NCI-H295R adrenocortical cell line as well. Our findings in culture medium underline that dexamethasone induces the secretion of* hsa-miR-27a* from adrenocortical cells (Figure 3). The molecular mechanism of* hsa-miR-27a* secretion in NCI-H295R cells and its interaction with dexamethasone, however, awaits further studies. In a rat model, ACTH-treatment also suppressed tissue* miR-27a* expression, but dexamethasone did the same. This discrepancy might be related to species differences; moreover, the expression of tissue and circulating microRNAs can be different [11].

The* in vitro* and* in vivo* action of dexamethasone on* hsa-miR-27a* expression is similar, since it upregulated* hsa-miR-27a* expression in both NCI-H295R adrenocortical cells* in vitro* (both cellular and secreted) and circulating* hsa-miR-27a in vivo*. The cellular origin of circulating microRNAs is, however, unclear, but these parallel changes in expression might raise the possibility of its partial adrenocortical origin. There are several data underlining the relevance of* hsa-miR-27a* in muscles, angiogenesis, adipogenesis and obesity, inflammation, immune response, lipid metabolism, atherosclerosis, and metabolic syndrome [23]. Circulating* hsa-miR-27a* has been raised as a biomarker for left ventricular contractility after acute myocardial infarction [24] and hypertrophic cardiomyopathy [25], and it was found to be underexpressed in early stage non-small cell lung cancer [26].

All these tissues are targets for glucocorticoid actions mediated via the glucocorticoid receptor. Since dexamethasone treatment also altered* hsa-miR-27a* expression in the NCI-H295R adrenocortical cell line, it might be hypothesized that this microRNA may be also regulated via the glucocorticoid receptor. As the transcription of* hsa-miR-27a* is made by RNA Polymerase II [27] it would be interesting to test whether a functional glucocorticoid response element is present in the* hsa-miR-27a* promoter (by* in silico* prediction, a glucocorticoid response element can be localized within the* hsa-miR-27a* promoter (data not shown)).

*hsa-miR-27a* has been shown to downregulate myostatin expression that is a major growth factor implicated in muscle development and muscle atrophy. Increased myostatin expression was associated with muscle wasting [28].* miR-27a* and myostatin appear to be involved in an autoregulatory loop as myostatin increases* miR-27a* expression via* SMAD3* and* miR-27a* in turn inhibits myostatin expression in a murine model [28]. As glucocorticoids inhibit the transcriptional activation of SMAD3 [29], administration of dexamethasone might interfere with the myostatin-*SMAD3*-*miR-27a* loop at multiple points. The ACTH-induced downregulation of circulating* hsa-miR-27a* might also be relevant, for example, in ACTH-dependent Cushing's syndrome. The overall effects of these actions on myostatin,* miR-27a,* and SMAD3 would be difficult to predict at present, but these findings might be implicated in the pathogenesis of glucocorticoid-induced muscle atrophy characteristic for hypercortisolism.

Levels of circulating* hsa-miR-27a* have been found to be strongly associated with fasting glucose levels and type 2 diabetes mellitus [30]. Since glucocorticoids are involved in the pathogenesis of insulin resistance [31], these findings raise the possibility that ACTH- and glucocorticoid-induced changes in* hsa-miR-27a* expression might be relevant in the pathogenesis of various diseases, and most of all in hypercortisolism, but further studies are needed to establish the pathological relevance of these alterations.

## 5. Conclusions

By analyzing the expression of selected microRNAs based on literature data, we have established that* hsa-miR-27a* is significantly downregulated by ACTH and induced by dexamethasone-treatment* in vivo*. We have also observed the* in vitro* induction of secreted* hsa-miR-27a* in adrenocortical NCI-H295R cells by dexamethasone. The expression of* hsa-miR-483-5p* proposed as a biomarker of ACC was not affected by hormonal treatments that underlines its applicability as a potential diagnostic test in the preoperative diagnosis of ACC. These data together highlight again that microRNAs are present in the circulation, and some of these are targets for hormone actions, and similarly to the hormone concentration measurement, strict preanalytical and analytical conditions should be followed before sampling.

## Figures and Tables

**Table 1a:** (a) Dexamethasone test

Patient number	Gender F/M	Age (year)	Disease/indication for testing	Baseline plasma cortisol (*μ*g/dL)	Cortisol after 1 mg Dex (*μ*g/dL)
1	F	73	Obesity	12.6	1.6
2	F	30	Obesity, hirsutism	19.5	0.5
3	M	65	Obesity	10.7	0.9
4	M	45	Hypertension	17.4	0.9
5	F	61	Obesity, hypertension	9.4	1.6
6	F	68	Adrenal incidentaloma	13.5	1.8
7	M	65	Adrenal incidentaloma	25.6	1.7
8	M	68	Adrenal incidentaloma	19.4	1.7
9	M	59	Adrenal incidentaloma	17.6	1.8
10	F	20	Obesity	20.0	1.3

**Table 1b:** (b) Tetracosactide test

Patient number	Gender F/M	Age (year)	Disease/indication for testing	Baseline plasma cortisol (*μ*g/dL)	Cortisol after 250 *μ*g tetracosactide (*μ*g/dL)
1	F	30	Sec. amenorrhea	14.4	35.8
2	F	46	Suspicion for AI	6.6	20.7
3	F	36	Weakness	13.4	31.4
4	F	23	Raromenorrhoea	16.3	33.9
5	F	36	Infertility	7.2	29.5
6	F	37	Sec. amenorrhea	14.2	35.4
7	F	34	Raromenorrhoea	26.0	35.7
8	F	23	Hirsutism	16.0	32.3
9	M	60	Suspicion for AI	15.4	28.0
10	F	23	Infertility	11.0	34.4

AI: adrenal insufficiency.
